# Novel Recurrent Cytogenetic Abnormalities Predict Overall Survival in Tetraploid/Near-Tetraploid Myelodysplastic Syndrome and Acute Myeloid Leukemia

**DOI:** 10.3390/cancers17081277

**Published:** 2025-04-10

**Authors:** Matthew R. Avenarius, Zachary B. Abrams, Ling Guo, James S. Blachly, Cecelia R. Miller, Nyla A. Heerema, Guilin Tang, Kevin R. Coombes, Lynne V. Abruzzo

**Affiliations:** 1 Department of Pathology, The Ohio State University Wexner Medical Center, Columbus, OH 43210, USA; matthew.avenarius@osumc.edu (M.R.A.); lin.guo@ohiohealth.com (L.G.); cecelia.miller@osumc.edu (C.R.M.); nyla.heerema@osumc.edu (N.A.H.); 2 Institute for Informatics, Washington University in St. Louis, St. Louis, MO 63110, USA; abramsz@wustl.edu; 3 Division of Hematology, Department of Internal Medicine, The Ohio State University Wexner Medical Center, Columbus, OH 43210, USA; james.blachly@osumc.edu; 4 Department of Hematopathology, The UT M.D. Anderson Cancer Center, Houston, TX 77030, USA; gtang@mdanderson.org; 5 The Georgia Cancer Center at Augusta University, Augusta, GA 30912, USA; kcoombes@augusta.edu

**Keywords:** acute myeloid leukemia, myelodysplastic syndrome, tetraploidy, cytogenetics

## Abstract

Background/Objectives: Tetraploidy (4n = 92 chromosomes) and near-tetraploidy (81–103 chromosomes) (T/NT) are uncommon cytogenetic events in MDS/AML (~1%). Abnormalities reported to be associated with T/NT MDS/AML include −5/del(5q), −7/del(7q), +8, and +21. However, other clinically relevant abnormalities likely remain “hidden” in long strings of ISCN cytogenetic nomenclature when evaluated visually. To date, no studies have had the statistical power and a computational method to identify novel recurrent abnormalities associated with the T/NT karyotype or overall survival (OS). Methods: Using CytoGPS, a bioinformatic tool we developed, we converted karyotypes from a combined cohort of 75 T/NT MDS/AML cases from two institutions into a binary Loss–Gain–Fusion model, which is analyzable using computational methods. Results: On univariate analyses, age as a continuous variable (*p* = 0.032), prior treatment (*p* = 0.011), and cohort (*p* = 0.025) were associated with OS; age ≥ 60 years (*p* = 0.316), gender (*p* = 0.916), karyotypic complexity (*p* = 0.175), time from diagnosis to T/NT karyotype identification (*p* = 0.419), and clone size (*p* = 0.316) had no effect. Univariate analyses of karyotypes demonstrated that −5, −16, −18, del(11)(p15.1p15.4), del(13)(q12.11q22.3), and +8 were associated with poorer OS (unadjusted *p* < 0.05). Conclusions: Using the results of univariate analyses to build multivariate models of OS, the best predictor of OS was the presence of any one of these six cytogenetic abnormalities.

## Simple Summary

Myelodysplastic syndromes (MDSs) and acute myeloid leukemias (AMLs) are malignant neoplasms that originate from myeloid progenitor cells in bone marrow. In patients with MDS/AML, recurrent chromosomal abnormalities identified by karyotyping, the practice of visually examining and recording chromosomal abnormalities, are among the most important markers of prognosis and are used to guide treatment. Karyotypes are recorded as text written in the International System for Human Cytogenetic Nomenclature (ISCN). To identify new prognostically important chromosomal abnormalities that remain “hidden” in long strings of ISCN nomenclature, we developed CytoGPS, a computer platform that unlocks the potential for large-scale, comparative analysis of karyotypic data. Patients whose MDS/AML cells have tetraploid karyotypes, that is, twice the normal number of chromosomes, tend to have many additional karyotypic abnormalities and a poor prognosis. Using CytoGPS to analyze karyotypes from 75 patients, we identified six additional chromosomal abnormalities that were associated with poorer overall survival.

## 1. Introduction

Myelodysplastic syndromes (MDSs) and acute myeloid leukemias (AMLs) are clonal malignant neoplasms that originate from myeloid progenitors and are characterized by dysplasia, ineffective hematopoiesis, and variable numbers of blasts. Cytogenetic characterization of these malignancies, along with the results of other hematological and morphological studies, is integrated into the World Health Organization (WHO) Classification, which is used to guide clinical management [1]. Tetraploidy (4n = 92 chromosomes), a doubling of the normal diploid chromosomal complement (2n), is an uncommon finding in a variety of cancers [2]. In myeloid malignancies, tetraploidy and near-tetraploidy (81-103 chromosomes) (T/NT) are uncommon cytogenetic events (~1%) of unclear clinical significance [3,4,5,6,7]. Most MDS/AML patients with T/NT are older adults with a male predominance [3,5,7,8]. In a recent series, karyotypic complexity and prior exposure to chemotherapy were associated with a poorer outcome [3]. However, T/NT MDS/AML patients who receive allogeneic hematopoietic stem cell transplantation may have a better outcome than those treated solely with chemotherapy [3,5].

Defects in two major cellular processes have been shown to lead to the formation of tetraploid cells. First, mitotic slippage occurs when spindle assembly checkpoint proteins delay chromosomal segregation due to improper chromosome orientation without simultaneously delaying the cell cycle. Second, chromosome(s) that remain at the spindle center after segregation may disrupt cellular division [9]. The tetraploid daughter cells are genomically unstable and promote transformation, as seen in other hematological malignancies, such as chronic lymphocytic leukemia and acute lymphoblastic leukemia [10,11].

Recurrent cytogenetic abnormalities associated with T/NT MDS/AML cases include −5/del(5q), −7/del(7q), +8, and +21 [3,5,6,7]. However, other recurrent, potentially clinically relevant, and actionable abnormalities likely remain “hidden” in long strings of cytogenetic nomenclature that are difficult to discern by simple visual inspection of karyotypes. In a previous proof-of-principle study, we applied Cytogenetic Pattern Sleuth (CytoGPS; http://cytogps.org (version 1.0.21)) [12], a publicly available bioinformatic tool that we developed, to all 4968 chronic myeloid leukemia karyotypes from the Mitelman Database of Chromosome Aberrations and Gene Fusions in Cancer (https://mitelmandatabase.isb-cgc.org/, (accessed on 2 July 2019)) and identified the co-occurrence of t(9;22)(q34;q11.2) with trisomy 21 [13], which has subsequently been shown to confer an increased risk of progression [14]. In the current study, we applied CytoGPS to a combined cohort of 75 T/NT MDS/AML cases and have identified novel recurrent cytogenetic abnormalities that affect overall survival (OS) in these patients.

## 2. Materials and Methods

### 2.1. Case Selection

We queried the files of the Cytogenetics Laboratory of The James Cancer Center at The Ohio State University (OSU) for myeloid neoplasms with a tetraploid or near-tetraploid (T/NT) karyotype. We identified 37 cases (1.8%) out of 2039 patients (treatment-naive and previously treated) seen between June 2002 and August 2018. As defined by the US Department of Health and Human Services, Office for Human Research Protections, this study was approved by The Ohio State University Cancer Institutional Review Board (2019C0202). For the University of Texas M.D. Anderson Cancer Center (MDACC) cohort of 38 T/NT AML cases, we extracted the clinical data and karyotype data from their Table 1 and Table 2, which were published previously [3].

### 2.2. Hematopathology and Conventional Cytogenetic Analyses

Bone marrow aspirate smears and core biopsies were reviewed by a hematopathologist and classified according to the WHO Classification system [1]. Conventional cytogenetic analyses were performed on metaphase cells using standard techniques, and results were reported using the International System for Human Cytogenomic Nomenclature (ISCN) [4]. The term “complex karyotype” is generally defined as a karyotype with greater than or equal to three unrelated structural and/or numerical abnormalities [15]. The term “complex karyotype” used to describe the T/NT cases in this study refers to karyotypes that contain greater than or equal to three unrelated structural and/or numerical abnormalities in a karyotype that contains two pairs of sex chromosomes (XXXX or XXYY) and four copies of each autosome.

### 2.3. CytoGPS

Karyotypes were processed using CytoGenetic Pattern Sleuth (CytoGPS; http://cytogps.org), a publicly available web-based tool that converts ISCN-formatted karyotypes into a binary computational data model referred to as the Loss–Gain–Fusion (LGF) model [12]. CytoGPS was implemented using ANother Tool for Language Recognition (ANTLR, version 4), a formal lexer and parser generator [16]. CytoGPS uses ANTLR to divide karyotypes into simpler units defined by the ISCN grammar; these units are then mapped to cytobands as binary indicators of the absence or presence of each of the three kinds of abnormalities: loss (L), gain (G), or fusion (F). Because the current version of CytoGPS cannot parse long-form ISCN nomenclature, karyotypes that contained long-form notation were rewritten in short form before CytoGPS analysis.

### 2.4. Statistical Analyses

The frequency of cytogenetic abnormalities, genome-wide or per chromosome, was visualized using version 1.1.1 of the RCytoGPS R package [17]. To compare abnormality rates between two cohorts (OSU versus MDACC, or T/NT versus non-T/NT), we applied a z-test of two proportions [18] at each cytoband for each of loss, gain, and fusion, followed by a correction for multiple testing. To compare clinical covariates between cohorts, we used standard *t*-tests for continuous variables and chi-squared tests for categorical variables. To test for associations between cytogenetic features and clinical outcomes (specifically, overall survival, OS), we performed univariate analyses using the Cox proportional hazards model with the (log-rank) score test. Kaplan–Meier plots were used to visualize the results of univariate modeling. Abnormalities that performed well in univariate analyses were selected for combined (clinical and cytogenetic) multivariate analyses to determine the best predictive model of OS in T/NT MDS/AML. We performed feature selection using a step-wise method based on the Akaike Information Criterion (AIC).

## 3. Results

### 3.1. Clinical Findings

The clinical and cytogenetic data are summarized in Table 1 and Table 2, respectively. There were 37 patients, 24 men (65%) and 13 women (35%), of median age 66.7 (range, 24–89 years). The diagnoses were acute myeloid leukemia (AML, 28 patients, 76%), myelodysplastic syndrome (MDS, 5 patients, 14%), acute undifferentiated leukemia (AUL, 3 patients, 8%), 1 of whom presented with myeloid sarcoma and histiocytic sarcoma (1 patient, 3%). The most common diagnosis was AML with myelodysplasia-related changes (AML-MRC) (21 patients, 57%) [8]. Thirty-four patients received intensive chemotherapy for AML, and seven also underwent allogeneic stem cell transplantation (SCT); three patients declined treatment. Twenty-seven patients (77%; cases 1–8, 11–14, 18–20, 22–25, 27–29, 32–35, 37) presented with a T/NT karyotype at MDS/AML diagnosis and seven (19%; cases 9, 10, 15, 21, 26, 31, 36) developed a T/NT karyotype after chemotherapy; data were unavailable for three patients (cases 16, 17, 30) (Table 1). Twenty-nine cases (83%; cases 1–8, 11–16, 18–24, 26, 28–32, 35, 37) showed complex karyotypes, and six were non-complex (17%; cases 9, 10, 17, 25, 27, 36); the karyotypic complexity of two cases (cases 33, 34) was unevaluable because the karyotypes were incomplete (inc), i.e., poor morphology precluded a complete cytogenetic analysis (Table 2). By conventional karyotypic analysis, 17 cases (46%; cases 1, 4, 7, 8, 10, 13, 16, 19, 20, 22, 28–32, 35, 37) showed a pseudodiploid clone that was cytogenetically related to the T/NT clone(s); the percent of abnormal T/NT metaphases ranged from 10% to 90% (median, 30%). Nineteen cases (51%; 2, 3, 5, 6, 9, 11, 12, 14, 15, 17, 18, 21, 23–25, 27, 33, 34, 36) showed no evidence of a pseudodiploid clone; the percent of abnormal metaphases that were T/NT ranged from 5% to 100% (median, 85%). In one case (case 26) the pseudodiploid clone was cytogenetically unrelated to the T/NT clone. For the eight patients who received chemotherapy before detection of a T/NT clone (cases 9, 10, 15, 21, 26, 30, 31, 36), three (cases 10, 30, 31) showed a cytogenetically related pseudodiploid clone, and five (cases 9, 15, 21, 26, 36) showed no evidence of a cytogenetically related pseudodiploid clone.

We next compared the clinical features between the OSU (n = 37) and MDACC (n = 38) cohorts (Supplemental Table S1). We found no difference in mean age (OSU, mean 66.7 yrs, range 24–89 years; MDACC, mean 61.3 yrs, range 21–88 years; *p* = 0.17), gender (OSU, 13 women, 24 men; MDACC, 12 women, 26 men; *p* = 0.93), or karyotypic complexity (OSU, 6 non-complex, 29 complex; MDACC, 10 non-complex, 28 complex; *p* = 0.51). However, we found statistically significant differences in prior treatment (OSU, 8/37 received prior therapy; MDACC, 19/38 received prior therapy; *p* = 0.03), the interval between diagnosis of MDS/AML and the identification of the T/NT clone (OSU, mean 5.36 months, range 0–84; MDACC, mean 12.1 months, range 0–132 months; Cox proportional hazards score test = 5.52, *p* = 0.0203), and the proportion of T/NT to non-T/NT clones for each case (OSU, mean 52%, range 5–100%; MDACC, mean 38%, range 15–90%; *p* = 0.007). Patients in the OSU cohort were less likely to have received prior treatment at the time the T/NT clone was identified than those in the MDACC cohort (OSU 27 treatment-naïve, 8 prior treatment; MDACC 19 treatment-naïve, 19 prior treatment; *p* = 0.03), likely reflecting referral patterns.

### 3.2. Recurrent Cytogenetic Abnormalities in T/NT MDS/AML Identified by CytoGPS

We next applied CytoGPS to identify recurrent cytogenetic abnormalities associated with the T/NT karyotype in the individual cohorts. We defined recurrent abnormalities as those present at a minimum frequency of 20%. This arbitrary cut-off was chosen to identify relatively common recurrent abnormalities that might be missed by visual inspection of highly complex karyotypes, but to reduce the detection of sporadic abnormalities. (The complete data are available in Supplemental Table S2). In the OSU cohort, CytoGPS detected the loss of chromosomes 2, 5, 7, 16, and 17 and partial deletions including del(3)(p13p26.3), del(9)(q13q34.3), del(11)(11p15.1-p15.4), and del(12)(q24.31q24.33). In the MDACC cohort, CytoGPS detected the loss of chromosomes 3, 4, 5, 7, 12, 15, 16, 17, and 18 and partial deletions including del(6)(q21.1q21.3), del(9)(p22.1p24.3), del(9)(q22.1q34.3), del(11)(p15.1p15.5), del(11)(q21q25), del(13)(q12.11q22.3), and del(20) (q11.21q13.33). In the OSU cohort, we detected a gain of chromosome 8 and almost all of chromosome 1 except for 1q11.1-21.3. While a gain of chromosome 8 was detected in the MDACC cohort, CytoGPS also identified gains of chromosomes 13 and 22. Thus, at a minimum frequency of 20%, both groups showed a loss of −5, −7, −16, −17, del(9q), del(11p), and +8 associated with the T/NT karyotype.

**Figure 1:**
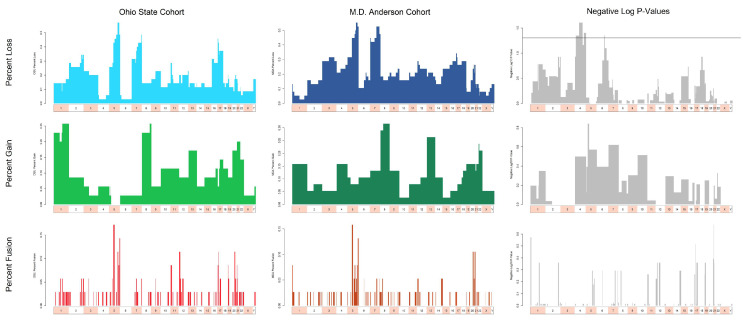
Comparison of abnormalities in T/NT clones between the OSU and MDACC cohorts. The top row shows losses (blue), the middle row shows gains (green), and the bottom row shows fusions (red). The left column displays the fraction of samples in the OSU cohort with an abnormality at the 850-cytoband resolution, and the center column shows the fraction of samples with an abnormality in the MDACC cohort. The right column displays the negative log_10_ *p*-values (gray) for each cytoband from a test of two proportions comparing OSU to MDACC; higher bars correspond to smaller *p*-values. The horizontal line in the upper right panel marks a nominal (unadjusted) *p*-value of 5%, which becomes non-significant after correction for multiple testing. All *p*-values for the middle right and lower right panels fail to reach the nominal *p*-value of 5%.

To improve the statistical power of our study, we determined that we could pool the karyotypes from both cohorts for subsequent analyses by applying the statistical test of two proportions to the CytoGPS analyses of the individual cohorts (Figure 1). Based on a nominal *p*-value cutoff of *p* < 0.05, the MDACC cohort showed the following recurrent deletions: del(4)(p15.33q28.3), del(4)(q32.1q35.2), and del(6)(q21.1q21.3), which were slightly more common than those seen in the OSU cases, comprising a total of 42 cytobands out of 868 total cytobands (4.8%, ~850 band level). However, these differences were not statistically significant after accounting for multiple testing. No other differences in the frequency of cytogenetic abnormalities were statistically distinguishable (Supplemental Table S2, Columns H–P). Thus, we performed subsequent analyses on the combined dataset of 75 cases.

### 3.3. Cytogenetic Abnormalities Over- and Under-Represented in T/NT Compared to Non-T/NT Karyotypes

**Figure 2:**
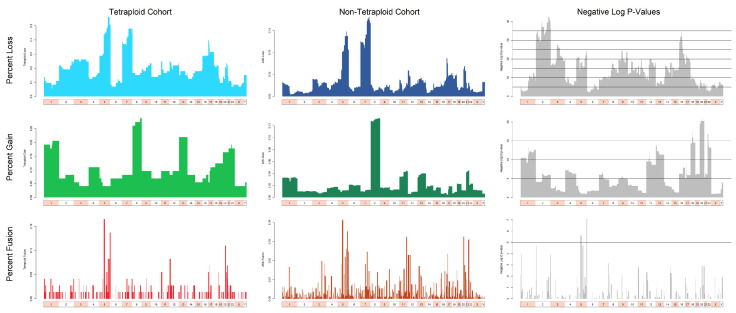
Comparison between abnormalities found in T/NT and non-T/NT cohorts. The top row shows losses (blue), the middle row shows gains (green), and the bottom row shows fusions (red). The left column displays the fraction of samples with an abnormality in the T/NT cohort at 850-cytoband resolution. The center column shows the fraction of samples with an abnormality in the non-T/NT cohort. The right column displays the negative log_10_ *p*-values (gray), for each cytoband, from a test of two proportions comparing T/NT to non-T/NT; higher bars correspond to smaller *p*-values. The horizontal lines in the panels in the right column indicate nominal *p*-values of 10^−5^, 10^−10^, 10^−15^, etc.

To identify recurrent abnormalities that distinguished T/NT MDS/AML from pseudodiploid/near diploid AML cases, we applied CytoGPS separately to the combined T/NT cohort (n = 75) and to a cohort of diploid/near diploid AML cases (n = 1872) obtained from the files of OSU. We then used a test of two proportions to find statistically significant differences in the proportions of abnormalities between the two cohorts (Figure 2). We found numerous chromosomal abnormalities significantly overrepresented (*p* < 10^−15^) in the T/NT cohort, including the loss of chromosomes 2, 3, 9, 16, and 17 and partial deletions: del(5)(p11p15.33), del(5)(q11.1q15), del(5)(q31.1q35.3), del(8)(q11.1q12.3), del(10)(q11.1q21.3), del(11)(p15.115.4), del(11)(q11q12.3), del(12)(q11q23.3), del(12)(q24.21q24.33), del(14)(q11.1q21.3), and del(14)(q32.11q32.33). Additionally, we detected gains in all cytobands of chromosome 20 and partial gains of 18q (18q11.1-q12.3) and 22p (22p11.2-p13). We identified no fusions as significantly overrepresented in the T/NT cohort, nor any abnormalities that were underrepresented in the T/NT cohort. Thus, T/NT MDS/AML cases contain a wider range of numerical and structural cytogenetic abnormalities than pseudodiploid/near-diploid cases.

### 3.4. Univariate and Multivariate Models of Overall Survival

**Figure 3:**
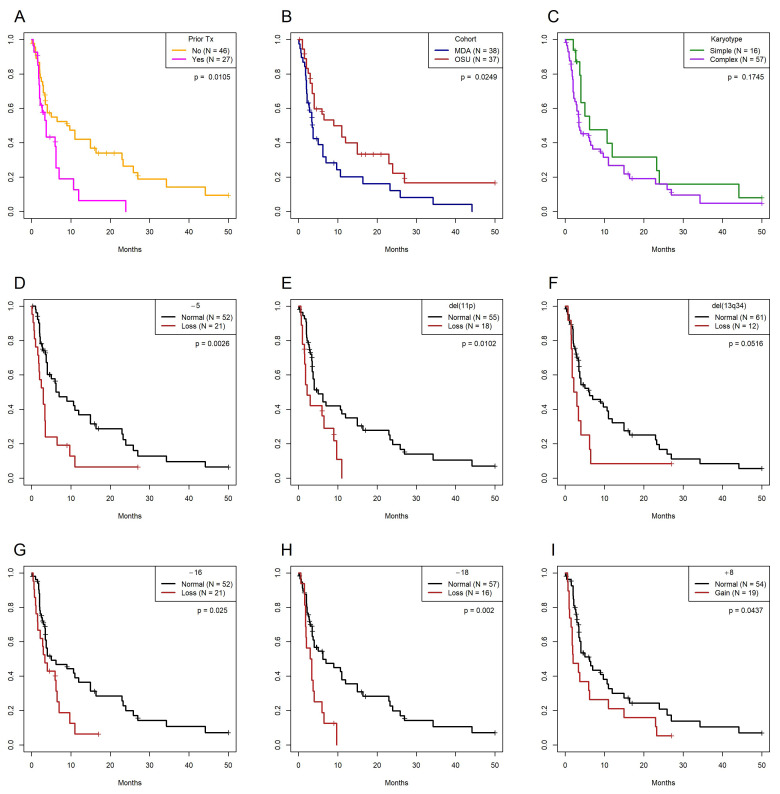
Kaplan–Meier plots for significant categorical factors predicting overall survival. (**A**) Prior therapy. (**B**) Institution where the patient was treated (OSU = Ohio State University James Cancer Center, MDA = M.D. Anderson Cancer Center). (**C**) Simple (<3 abnormalities) or complex (≥3 abnormalities) karyotype. (**D**) Loss of chromosome 5. (**E**) Loss of 11p15.1-p15.4. (**F**) Loss of 13q34. (**G**) Loss of chromosome 16. (**H**) Loss of chromosome 18. (**I**) Gain of chromosome 8.

To develop a multivariate model of OS, we first performed separate univariate analyses on the combined clinical and karyotypic data from the T/NT patients (Table 3). Clinical variables included in the univariate analysis were age (continuous and categorical), gender, prior chemotherapy treatment, karyotypic complexity, cohort, interval between diagnosis of MDS/AML and identification of the T/NT clone, and T/NT clone size. Age as a continuous variable (*p* = 0.032), prior treatment (*p* = 0.011; Figure 3A), and cohort (*p* = 0.025; Figure 3B) correlated with OS. In contrast, age ≥ 60 years (*p* = 0.316), gender (*p* = 0.916), karyotypic complexity (*p* = 0.175; Figure 3C), interval between diagnosis and identification of the T/NT karyotype (*p* = 0.419), and T/NT clone size (*p* = 0.316) had no effect. Univariate analyses of the CytoGPS data demonstrated that losses of chromosomes 5, 16, and 18, deletions of 11p and 13q, and a gain of chromosome 8 were significantly associated with poorer OS (unadjusted *p* < 0.05) (Figure 3D–I, Supplemental Figure S1, and Supplemental Table S2, Columns Z–AB).

We used the results of the univariate analyses to build three multivariate models of OS (Supplemental Table S3). In the first model, we used all six cytogenetic factors with unadjusted *p* < 0.05 (−5, −16, −18, del(11)(p15.1p15.4), del(13)(q12.11q22.3), +8), without accounting for multiple testing, and all four clinical factors with *p* < 0.20 (age, prior treatment, cohort, and karyotypic complexity) as potential prognostic factors. The cutoff of *p* < 0.20 retained karyotypic complexity as a possible factor, since it is a known prognostic indicator in AML [19] and was previously reported to be an important prognostic factor in the MDACC cohort [3]. Applying stepwise selection and the Akaike Information Criterion (AIC), we found that the four clinical variables (age, prior treatment, cohort, and karyotypic complexity) should be included in a purely clinical multivariate model. In the second model, we applied the same approach to the six significant chromosomal regions identified in the univariate analysis. This approach identified −5 and −16, and +8, as the cytogenetic abnormalities that survived the stepwise analysis. Finally, in the third model, we applied the same method to construct a multivariate model that combined the four clinical variables and six cytogenetic variables. Starting with these 10 variables, the best model by the AIC retained all four clinical variables along with −5 and del(11)(p15.1p15.4). Further, we found that −5 was the cytogenetic event most strongly correlated with OS among T/NT patients.

Interestingly, the same statistical methods found different important cytogenetic features in the second and third models described above. In the second model, which considered only the six cytogenetic variables, −5 and −16 and +8 were statistically significant. In the third model, which considered both cytogenetic and clinical variables, only −5 and del(11)(p15.1p15.4) were statistically significant. We hypothesized that the difference between the second and third models occurred because the information provided by the cytogenetic abnormalities was redundant with one or more of the clinical variables. To examine this possibility, we tested the association between each cytogenetic variable and each clinical variable. Because all cytogenetic variables are binary, i.e., the presence or absence of a specific abnormality, we used *t*-tests for continuous clinical variables and chi-squared tests for categorical clinical variables. The only significant associations that we found were between cytogenetic variables and karyotypic complexity, which was strongly associated with −5 (*p* = 0.016), −16 (*p* = 0.012), −18 (*p* = 0.045), and del(11)(p15.1p15.4) (*p* = 0.027). The associations with +8 (*p* = 0.098) and del(13)(q12.11q22.3) (*p* = 0.114) were weak. These results suggest that the cytogenetic variables tend to co-occur with each other and with complex karyotypes. Thus, it is not surprising that we found different cytogenetic abnormalities that were prognostically important in models 2 and 3.

**Figure 4:**
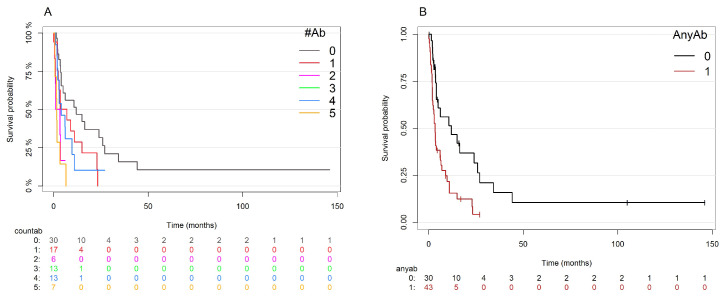
Kaplan–Meier plots showing effects on overall survival of two ways to combine the six cytogenetic abnormalities of interest. (**A**) The count of the number of abnormalities present in each patient. (**B**) A binary indicator of the presence of any one of the six abnormalities.

Because the six cytogenetic abnormalities appeared to be nearly interchangeable in the multivariate models to predict OS, we tested two ways to combine the cytogenetic variables. First, we counted the number of these six abnormalities that were present in each patient (Figure 4A). This count variable was statistically significant (score test = 15.62, *p* = 8 × 10^−5^). However, the main difference appeared to arise from the presence of any one of these abnormalities compared to having none of them. Therefore, we tested a logical binary variable measuring the presence of at least one of the six abnormalities. This variable was also statistically significant (score test = 9.8, *p* = 0.002; Figure 4B). Moreover, when we compared “any of the 6” to “complex karyotype”, either alone or in the context of other clinical or cytogenetic variables, complex karyotype was removed from the model while “any of the 6” was retained. In other words, having any one of these six cytogenetic abnormalities was a better predictor of OS than complex karyotype in this cohort of T/NT MDS/AML cases.

## 4. Discussion

Cytogenetic abnormalities remain important markers for risk stratification in patients with MDS and AML. One of the most commonly used classification systems, developed by the European LeukemiaNet, divides AML into three risk categories (favorable, intermediate, and adverse) based on the results of cytogenetic and molecular diagnostic testing [19]. Cytogenetic abnormalities in the adverse risk category include −5/del(5q), −7, and −17/abn(17p), inv(3)(q21.3q26.2) and t(3;3)(q21.3;q36.2), and complex karyotype. Because T/NT is uncommon in myeloid malignancies, there are no studies that have had both statistical power and the use of a computational method to identify novel recurrent abnormalities associated with the T/NT karyotype or their impact on OS.

Consistent with previous studies, we found that the vast majority of T/NT MDS/AML patients were older adults with a marked male predominance and a dismal prognosis, with an OS of only months after the identification of a T/NT clone [3,5,7,8]. The small proportion of patients who remained alive in CR were younger and had received intensive chemotherapy followed by allogeneic stem cell transplantation [3,5]. In the current study, about three-quarters of cases showed a T/NT clone at diagnosis, and a similar proportion showed a complex karyotype. About one-half of the cases showed a cytogenetically related pseudodiploid clone, indicating that the T/NT clone likely arose with the duplication of the pseudodiploid clone. Of these cases, about one-half of the patients had received prior chemotherapy, and the other half were treatment-naïve, suggesting that duplication events are not necessarily therapy-related.

AML cases with core-binding factor translocations, i.e., inv(16)(p13.1q22), t(16;16)(p13.1;q22), and t(8;21)(q22;q22.1), are classified in the “favorable” risk category regardless of the presence of other karyotypic abnormalities [15,19]. Our combined cohort contained a single case of T/NT AML with t(8;21); we identified 17 additional cases of T/NT AML with t(8;21) in the literature [6,20,21,22,23,24,25,26]. In contrast to most T/NT MDS/AML cases, seven arose in children—six girls and one boy. In all cases, the T/NT clone contained two copies of the t(8;21), and all but two contained a pseudodiploid clone with a single copy of the t(8;21), suggesting that T/NT clones with t(8;21) arose with the duplication of a pseudodiploid/near-diploid clone. The overall survival of T/NT AML with t(8;21) is poor, with a median survival of 9.5 months [6,20,21,22,23,24,25,26]. Although the numbers are small, our findings suggest that T/NT with t(8;21) may be better classified as an “adverse” risk category.

One goal of our study was to identify recurrent cytogenetic abnormalities in T/NT karyotypes that may have escaped detection with a simple visual inspection of long strings of cytogenetic nomenclature. To improve the statistical power of this study, we first determined that we could combine data from our patient cohort with a recently published, well-documented cohort of patients treated at the MDACC. Thus, the combined dataset included a total of 75 patients with T/NT MDS/AML. In our initial analysis comparing T/NT MDS/AML to pseudodiploid/near-diploid AML cases, we found that T/NT MDS/AML cases contain a wider range of numerical and structural abnormalities, predominantly the loss of whole chromosomes and partial deletions, than pseudodiploid/near-diploid AML cases; we identified no fusions that were over-represented in the T/NT cases. Similar to previous studies, we identified −5, −7, −17, and +8 as recurrent abnormalities associated with T/NT MDS/AML [3,5,6,20]. We also identified −16, del(9q), and del(11p) as recurrent abnormalities.

Our major goal was to identify recurrent cytogenetic abnormalities in T/NT MDS/AML with prognostic impacts. To develop multivariate models of OS, we first performed separate univariate analyses on the combined clinical and karyotypic data. We identified three clinical variables (age, prior treatment, and cohort) and six cytogenetic abnormalities (−5, −16, −8, del(11p), del(13q), and +8) that correlated with OS. Because other studies have identified karyotypic complexity as correlating with OS, we included it in our multivariate models of OS. Similar to other studies, we found that patients with T/NT MDS/AML have a generally poor OS. However, our analysis yielded several unexpected results. Of the recurrent cytogenetic abnormalities associated with adverse risk in patients with pseudodiploid/near-diploid AML [19], only −5 was associated with a worse OS. We found no additional impact on OS of −7, −17, complex karyotype, or translocations associated with adverse risk. Further, any one of six recurrent cytogenetic abnormalities (−5, −16, −18, del(11)(p15.1p15.4), del(13)(q12.11q22.3), +8) in the presence of a T/NT karyotype portended a poorer OS. Trisomy 8, a common recurrent abnormality in a wide variety of myeloid neoplasms, is usually considered to confer an intermediate risk in patients with AML [19,27]. However, our findings suggest that +8 in T/NT MDS/AML may confer an adverse risk. Monosomy and partial deletions of chromosomes 16 and 18 are rare abnormalities in myeloid and lymphoid malignancies, usually associated with complex karyotypes [28,29,30,31]. The downregulation of *TRADD* expression (16q22) and deletion of *CBFB* (16q22.1) in patients with myeloid neoplasms have been associated with poor prognosis [30,32]. Several candidate tumor suppressor genes have been identified on 18p [29]. Similarly, reports of 11p deletions in myeloid neoplasms are rare. The cytoband 11p15 is the site of *NUP98*, which is involved in rearrangements with a variety of partner genes in acute lymphoid and myeloid leukemias [https://www.omim.org/entry/601021?search=NUP98&highlight=nup98 accessed 2 January 2022]. Deletion of *NUP98*, reported in rare cases of AML, is of unknown prognostic significance [33]. While del(13q) is a recurrent cytogenetic abnormality with prognostic significance in a wide variety of lymphoid malignancies, it is uncommon in myeloid malignancies, and its prognostic significance is unclear [http://atlasgeneticsoncology.org/Anomalies/del13qID1096.html accessed 2 January 2022]. Recent studies have identified mutations in *TP53,* followed by *RUNX1* and *SRSF2,* as the most common in NT/T AML cases [8]. Unfortunately, because most cases in our cohort were collected before mutation analysis was performed routinely, we cannot assess the impact of prognostically important mutations in combination with cytogenetic abnormalities on OS.

## 5. Conclusions

In summary, we have applied a computational approach to identify novel recurrent cytogenetic abnormalities with prognostic significance in T/NT MDS/AML cases. Taken together with previous studies, our findings suggest that T/NT MDS/AML cases, including those with t(8;21), should be classified as adverse risk, regardless of karyotypic complexity. Finally, we would encourage investigators with access to large cytogenetics databases to apply the CytoGPS tool (http://cytogps.org/) to their data to determine if the results can be validated on an independent dataset.

## Figures and Tables

**Table 1:** Clinical Features.

Case	Age * (Yrs)/Gender	Interval ^†^ (Months)	Diagnosis	Prior Treatment	Treatment	T/NT Size ^§^ (%)	T/NT Persist. ^‡^	OS (Months)	Outcome
1	63/M	0	MDS-EB1	No	Chemo	21	No	2.5	DOD
2	65/M	0	AML-MRC	No	Chemo	95	No	3	DOD
3	89/M	0	Histiocytic sarcoma	No	No	20	NA	1	DOD
4	89/F	0	AML-MRC	No	Chemo	75	NA	1.5	DOD
5	84/M	0	AML-MRC	No	Chemo	85	Yes	15	DOD
6	78/F	0	AML-MRC	No	Chemo	74	Yes	2	DOD
7	67/M	0	MDS-EB1/ evolving AML	No	Chemo	25	Yes	11	DOD
8	85/M	0	AML-MRC	No	Chemo	35	NA	27	DOD
9	69/M	48	AML, NOS	Yes	Chemo	75	Yes	24	DOD
10	79/M	2.5	AML, NOS	Yes	Chemo	10	No	3	LFU—hospice
11	87/M	0	AML-MRC	No	No	90	NA	0.25	LFU—hospice
12	58/M	0	AML-MRC	No	Chemo	85	NA	3.5	DOD
13	70/F	0	Myeloid sarcoma/AUL	No	Chemo	90	No	4.5	LFU—hospice
14	57/M	0	AML-MRC	No	Chemo	100	No	15	DOD
15	72/M	84	AML, NOS	Yes	Chemo	25	Yes	2	DOD
16	61/M	0	AML-MRC	NA	Chemo	30	No	3.5	DOD
17	65/M	NA	AML-MRC	NA	Chemo	90	No	4	DOD
18	52/M	0	MDS-EB2	No	Chemo	95	NA	4	DOD
19	58/M	0	AML-MRC	No	Chemo	15	Yes	11	DOD
20	66/M	0	AML-MRC	No	Chemo	25	Yes	1	DOD
21	75/F	8	AML t(8;21)	Yes	Chemo	100	Yes	6	LFU
22	24/M	0	AML-MRC	No	Chemo, SCT	85	No	146	ANED
23	71/M	0	AML, NOS	No	Chemo	55	Yes	23	DOD
24	69/F	0	AML-MRC	No	Chemo, SCT	100	No	6.5	DOD
25	29/F	0	AUL	No	Chemo, SCT	13	No	105	ANED
26	62/F	21	AML-MRC	Yes	Chemo	85	Yes	1.5	DOD
27	77/M	0	MDS-MLD	No	No	35	NA	4	DOD
28	61/M	0	AML-MRC	No	Chemo, SCT	43	No	9	DOD
29	69/F	0	AML-MRC	No	Chemo	10	Yes	16	LFU
30	69/F	NA	AML-MRC	Yes	Chemo	20	Yes	1.5	LFU
31	78/M	4	AML-MRC	Yes	Chemo	70	Yes	6	DOD
32	69/M	0	MDS-EB2	No	Chemo	35	No	27	ANED
33	38/F	0	AUL	No	Chemo, SCT	22	No	19	ANED
34	46/M	0	AML, NOS	No	Chemo, SCT	22	No	21	ANED
35	82/F	0	AML-MRC	No	Chemo	60	Yes	3	DOD
36	61/F	20	AML inv(3)	Yes	Chemo, SCT	5	Yes	12	DOD
37	74/F	0	AML-MRC	No	Chemo	10	Yes	17	AWD

* Age when T/NT clone was identified. ^†^ Interval between diagnosis of AML/MDS and identification of the T/NT clone. ^§^ The size of the T/NT clone as a percentage of the cells in the sample. **^‡^** Persistence of T/NT clone in post-diagnostic sample(s). Abbreviations: M, male; F, female; NA, not available; MDS-EB1, myelodysplastic syndrome with excess blasts 1; AML-MRC, acute myeloid leukemia with myelodysplasia-related changes; AML, NOS, AML not otherwise specified; AUL, acute undifferentiated leukemia; AML t(8;21), AML with t(8;21)(q22;q22); MDS-EB2, myelodysplastic syndrome with excess blasts 2; AML inv(3), AML with inv(3)(q21;q26); Chemo, chemotherapy; SCT, allogeneic stem cell transplantation; T/NT, tetraploid/near-tetraploid; OS, overall survival; DOD, died of disease; LFU, lost to follow-up; ANED, Alive, no evidence of disease; AWD, alive with disease.

**Table 2:** Karyotypes.

Case	Complexity	Karyotype
1	C	44~49,XY,-2,-5,del(7)(q22),-19,+21,add(21)(p11.2)x2,+2-5mar[cp9]/ 47~49,sl,add(12)(q24.3)[cp3]/87~113,slx2[cp4]/46,XY[3]
2	C	86~92<4n>,XXYY,del(2)(p23p25)x2,-5,-5,-9,-9,add(11)(p15)x2,-13,-17,-17,+22, +mar1x2,+mar2x2,+mar3x2,dmin[cp19]/46,XY[1].ish dmin(CMYC+)
3	C	72~98<4n>,XYY,-X,+1,i(1)(q10),+8,+8,-11,+12,+12,+16[4,three w/nonclonal abnormalities]/46,XY[16,one is 4n]/nonclonal[1]
4	C	46,XX,del(5)(q13q33),del(20)(q11.2q13.1)[2]/77~89,idemx2,del(1)(q21),-2,-3, del(3)(p25),+8,add(10)(q24),hsr(11)(q23),hsr(11)(q23),-11,add(13)(q34),-16,-17,-17, -17,del(18)(q21),+19,+19,add(19)(p13),der(19)t(17;19)(q21;p13)x2,+20,+20,-del(20), -del(20),+mar[cp18]/46,XX[4]/nonclonal[1] .ish hsr(11)(amp MLL),hsr(11)(amp MLL)
5	C	83~88<4n>,XXYY,i(1)(q10),-2,-3,-7,-9,-17,i(17)(q10),+18[cp18]/46,XY[2]/4n[1]
6	C	85~90<4n>,XXXX,-8,-9,-9,-12,-17,i(17)(q10),+mar1x2,+mar2,+dmin[cp10]/ 81~89,idem,-2,-5,-5,-7[cp7]/46,XX[6]
7	C	45~50,XY,-5,der(7)t(5;7)(p14;p12)add(7)(q11.2),der(13;14)(q10;q10)c,+21,+mar1, +mar2,+mar3,+mar4,+mar5[cp8]/44,sl,add(4)(p16),add(12)(p12),add(19)(p13), -mar1,-mar4,-mar5[cp3]/85~87,slx2,+1,add(19)(p13)x2,-mar1,-mar1,-mar2,-mar3, -mar4,-mar5,+mar6[cp4]/45,XY,der(13;14)(q10;q10)c[1]. ish der(7)t(5;7)(D5S23:D5S721+,D7Z1+,D7S486-)
8	C	46,XY,t(9;22)(q34;q11.2)[11]/89-93,idemx2,+4,add(4)(q31)x2[7]/nonclonal Ph+[2]
9	N	94<4n>,XXYY,+13,+13[cp15]/46,XY[3]/nonclonal[2]
10	N	46,XY,del(6)(p23)[17]/90-92,sl,x2[2]/46,sl,add(19)(p13.3)[1]
11	C	90<4n>,XXYY,add(2)(q31),-7,-7[4]/90,sl,del(5)(q31q35)[cp14]/46,XY[2]
12	C	97,XXYY<4n>,+1,t(1;8)(q32;q24),der(1)t(1;8),-3,+4,del(5)(q31q35),del(7)(q10), +10,+12,add(12)(q13)x3,+21,+22[17]/nonclonal w/clonal abnormalities[3]
13	C	47,XX,+10[2]/94-99<4n>,XXXX,+X,+X(4),-2,-7(6),-9,+10,+10,+10,-16,+20(9), +der(?)t(?;1)(?;q25)x2,+mar1[cp18]
14	C	90<4n>,XX,-Y,-Y,del(5)(q14)[15]/93,idem,+8,+11,+13[5]
15	C	90<4n>,XX,-Y,-Y,add(7)(q36)x2[cp5]/46,XY[13]/nonclonal[2]
16	C	45~47,XX,del(3)(p13),del(5)(q11.2q35),add(17)(p13),-20,+mar1[cp5]/ 48,idem,-del(3)(p13),+add(20)(q11.2),+22,+mar2[cp8]/76~97,idemx2,-del(3)(p13)x2, -5,+add(20)(q11.2)x2,+22,+mar2x2[cp4]/120~134,idemx3[cp2]/46,XX[1]
17	N	92<4n>,XXYY,der(5)t(1;5)(q21;q31)[18]/46,XY[2]
18	C	71~83<4n>,XXYY,add(2)(q37),del(2)(q33q35),-7,-13,-13,-13,add(13)(p12),-14, add(14)(p13),-15,-16,-17,-18,-20,+21,idic(21)(p13),psu dic(21;13)(p11.2;p13),-22, +mar1,+mar2,+mar3,+mar4,+mar5[cp5]/74~83,idem,-add(13)[cp14]/46,XY[1]
19	C	46~48,X,-Y,dup(1)(q23q42),del(5)(q13q33),-7,del(7)(q11.2),+8, der(11)t(Y;11)(q11.2;p13),+20,add(20)(q11.2)x2,+mar1[cp3]/ 42,sl,add(3)(p25),-8,-12,-17,-add(20),-mar1[7]/42,sl,+1,-dup(1),-3,+7,-del(7),-8, del(12)(p11.2p13),-16,-mar1,+mar2[cp7]/88-90,sdl2x2,+mar3[cp3]
20	C	46,XY,-1,-5,+8,+11,add(11)(p11.2)x2,-17,der(17)t(1;17)(p22;p11.2),+mar[12]/ 77~79,idemx2,-2,-3,-8,-8,-add(11),-add(11),-12,-14,-16,-mar[cp5]/46,XY[3]
21	C	73~87<4n>,XXXX,-7,-7,t(8;21)(q22;q22)x2,-11,-14,-16,-20[cp20]
22	C	44,X,-Y,add(5)(q11.2),-9,-12,der(17;18)(q10;q10),+mar1,+mar2[2]/ 85,idemx2,-2,-3,-7[17]/nonclonal[1]
23	C	99~110<4n>,XXYY,+2,+3,+8,+10,+12,+13,+13,+13,+16,+18,+19,+20,+21,+22,+22,+mar1x2[cp8]/81~82<3n>,XY,-X,+Y,+2,+9,+10,+13,+13,+14,+14,+15,+15,+19,+20,+21,+22[cp3]/ 46,XY[9]
24	C	82~88<4n>XXXX,-1,t(3;11)(p13;q21),-5,del(5)(q13q31),dic(9;21)(q12;p13),-13,-13,-16, -17,-17,dic(17;21)(p13;p12),-18,-18,-18,+21,add(22)(p13),+der(?)t(?;1)(?;p13), +der(?)t(?;1)(?:q21),+mar1,+mar2[cp8]/82-88,sl,-der(11)t(3;11)[cp16] .ish t(3;11)(MLL+,MLL-)
25	N	94<4n>,XXXX,+13,+13[cp3]/46,XX[19]/4n[1]
26	C	92~95<4n>,XXXX,+2,+2,del(2)(q33q37)x3,+13,+13,-21[6]/90~95,sl,+2,del(2)[3]/90-95,sdl1,-21[5]/86~94,sl,-21[3]/46,XX,t(1;12)(p36.1;p13)[2]/46,XX[1]
27	N	92<4n>,XXYY[7]/46,XY[13]
28	C	45,X,-Y,del(5)(q13q33),add(14)(q24),-17,-19,+mar1,+mar2[cp9]/ 90,slx2,add(11)(p15)x2[cp10]/46,XY[4]
29	C	46,XX,add(7)(q36),-10,+mar[17]/92,slx2[2]/46,XX[1]
30	C	46,XX,del(5)(q13q35),add(7)(q11.2),-11,add(17)(p11.2),+20,del(20)(q11.2q13.3), dic(20;22)(q11.2;p11.2),+der(?)t(?;11)(?;q12),dmin[6,two w/nonclonal abnormalities]/45,sl,der(16)t(1;16)(p13;p11.2),-del(20)[6,two w/nonclonal abnormalities]/89,sdl1x2,+16,-der(16)t(1;16)x2[2]/ 90,sdl2,+1[2]/47,sl,+ider(?)(11qter->11q12::?::11q12->11qter)[2,one is 4n]/46,XX[2]
31	C	43,X,-Y,del(5)(q22q35),add(7)(q21),+8,add(9)(q13),-10,add(11)(p15), der(12)del(12)(p12p13)add(12)(q13),+15,add(15)(q22),-16,-17,-18,-19,add(20)(p12), +mar1[2,one w/nonclonal abnormalities]/ 82~85,slx2,idic(1)(q44),-del(7)x2,+17,+18[cp4]/75,sdl1,+1,+add(1)(q21),-dic(1;1)[cp3]/ 72~79,sdl2,+add(1)(q25),-add(1)(q21),-15[cp4]/69~75,sdl1,-15,-15,-21,-mar1[cp3]/ 46,XY[4]
32	C	43~51,XY,+1,+2,-3,-5,+6,-7,+8,del(9)(q13q22),add(12)(p13),+13,+14,+15,+15,-17,add(17)(p11.2),+18,der(20)(20pter->20q13.1::?::5p15.2->5p15.2::?),+21,+21, der(21)add(21)(p13)del(21)(q22),der(21)t(9;21)(q13;p13)x2,+22,+mar[cp4]/ 42~55,sl,add(3)(p13),+9,del(9)(q13q22),+11,-14,-22[cp5]/84,sdl1x2,-X,-1,-1,-2,-2, -del(3),add(4)(q21),-8,-9,-del(9),-10,-13,-13,-13,-add(17),-19, -der(21)add(21)del(21)[cp3]/51~81,sl,der(1)t(1;9)(p13;q13),-2,+8,del(9)(q13q22), -15,+19,+21,+der(21)add(21)del(21),+der(21)t(9;21)[cp4,2 are 4n]/ 82~84,slx2,-X,-2,-2,-4,+8,-12,-13,-22,-22[cp2]/46,XY[2] .ish add(12)(ETV6-),der(20)(D5S23:D5S721+,D20S108+),der(21)t(9;21)(RUNX1+)
33	Inc	91<4n>,XXXX,-17,inc[cp5]/46,XX[15]/nonclonal[3]
34	Inc	88~91<4n>,XXYY,inc[cp5]/46,XY[18]
35	C	47,XX,+10[1]/85,slx2,-5,-6,-7,-8,-10,-15,-16,-17,-18[7]/85,sdl1,-2,+r[5]/ 46,sl,dic(7;16)(p13;q12.1)[5]/46,XX[2]
36	N	90<4n>,XXXX,inv(3)(q21.3q26.2)x2[1]//46,XY[19]/nonclonal[1]
37	C	45,XX,der(5)t(5;16)(q13;q22),del(13)(q12q22),-16,add(17)(p13),der(17)del(17)(q11.2)del(17)(q11.2),add(22)(q13),+psu dic(?;17)(?::17q21->17q21::17p11.2->17q11.2::17q21->17q21::?)[18,one w/nonclonal abnormalities]/92,slx2[cp2].ish der(2)cryp ins(2;17)(TP53+),der(5)t(5;16)(CBFB+),add(17)(TP53+),psu dic(?;17)(TP53-,RARA+,D17Z1+,RARA+)

Of the 34 patients who elected to receive treatment, the T/NT clone persisted in 16 (47%) following treatment; data were unavailable for 7 patients (cases 3, 4, 8, 11, 12, 18, 27). On the last follow-up, 25 patients had died of disease at a median of 4 months after the identification of the T/NT clone (range, 1–27 months), 1 patient was alive with disease (case 37), 3 were discharged to hospice care, and 3 (cases 21, 29, 30) were lost to follow-up. Five patients were alive in complete remission (CR) (cases 22, 25, 32–34). All presented with the T/NT karyotype; four were younger than 60 years at presentation and had received SCT (cases 22, 25, 33, 34). C = complex karyotype, N = not complex, Inc = Incomplete.

**Table 3:** Univariate Analysis.

Factor		Number of Cases	Deaths	Median Survival (Range)	Coefficient	Hazard Ratio	Log-Rank Test	*p*-Value
Age in years (continuous)	NA	NA	NA	NA	0.019	1.019	4.59	0.032
Age in years (categorical)	<60	23	15	4 (3.4–∞)	NA	NA	NA	NA
	≥60	52	42	5 (3.4–10.7	10,433	1.542	1.954	0.162
Gender	Female	25	16	3.7 (3.4–∞)	NA	NA	NA	NA
	Male	50	41	6 (3.5–11)	0.031	1.032	0.011	0.916
Prior Rx	No	46	34	9 (3.5–23)	NA	NA	NA	NA
	Yes	27	21	3.7 (2–7)	0.743	2.102	6.541	0.011
Karyotype *	Simple	16	12	6.2 (4–∞)	NA	NA	NA	NA
	Complex	57	45	3.5 (3–9)	0.443	1.1557	1.843	0.175
Cohort	MDACC	38	32	3.5 (2.2–7)	NA	NA	NA	NA
	OSU	37	25	(4–24)	−0.598	0.55	5.032	0.025
Interval ^†^	NA	NA	NA	NA	0.004	1.004	0.654	0.419
T/NT Clone Size	NA	NA	NA	NA	0.005	1.005	1.007	0.316

Abbreviations: NA, not applicable; MDACC, M.D. Anderson Cancer Center; OSU, The Ohio State University. * OSU cases 33 and 34 were not included in this analysis because their karyotypes were incomplete. ^†^ Interval between diagnosis of MDS/AML and identification of the T/NT clone.

## Data Availability

All original data have been made available as an Excel file in Supplemental Table S2. The CytoGPS bioinformatic analysis tool is publicly available at http://cytogps.org.

## Supplementary Materials

The following supporting information can be downloaded at https://www.mdpi.com/article/10.3390/cancers17081277/s1, Supplemental Table S1. Cohort Comparison; Table S2. Excel file containing the complete results, by cytoband and abnormality type, of all statistical analyses; Table S3. Univariate and multivariate models of overall survival; Figure S1. Each of the six panels displays one of the six recurrent abnormalities identified as associated with overall survival in AML patients with tetraploid or near-tetraploid (T/NT) karyotypes. Each abnormality is defined by a cytogenetic event in a specific cytoband (loss of 5p14.2, gain of 8p12, loss of 11p15.3, loss of 13q34, loss of 16q22.1, or loss of 18q21.32). Each panel compares patients with that abnormality (to the left of the ideogram; orange for loss and green for gain) to patients without (on the right, in gray). Each half-panel shows the fraction of patients with the abnormality, but displays these data for the entire chromosome. For all but one abnormality, the loss or gain fails to extend to the entire chromosome for at most one sample. The exception is a deletion of 11p that only involves the telomeric portion of the p-arm.
