# Supplementary material for: Novel Recurrent Cytogenetic Abnormalities Predict Overall Survival in Tetraploid/Near-Tetraploid Myelodysplastic Syndrome and Acute Myeloid Leukemia
Source: Cancers (Basel). 2025 Apr 10;17(8):1277. doi: 10.3390/cancers17081277 (PMC12025582; doi:10.3390/cancers17081277)
Supplement: Supplementary file 1 [file cancers-17-01277-s001.zip › cancers-3507496-supplementary.pdf]

Supplemental Table S1: Cohort Comparison

Factor	OSU	MDACC	Statistic	p-value
Age (years) (mean; range)	66.7 (24-89)	61.3 (21-88)	t = -1.3936	p = 0.1678
Gender<br>Male<br>Female	 24<br>13	 26<br>12	χ^2 = 0.0067	p = 0.9349
Interval (months) (mean; range)	5.36 (0-84)	12.1 (0-132)	Score = 5.52	**p = 0.0203**
Prior Therapy<br>Yes<br>No	 8<br>27	 19<br>19	χ^2 = 4.6535	**p = 0.0310**
T/NT size (%) (mean; range)	52.16 (5-100)	38.03 (15-90)	t = 2.7592	**p = 0.0067**
Karyotype<br>Non-complex<br>Complex	 6<br>29	 10<br>28	χ^2 = 0.0440	p = 0.5071

Abbreviations: OSU, The Ohio State University; MDACC, M.D. Anderson Cancer Center

†Time from diagnosis of AML/MDS until identification of the T/NT clone. The interval was analyzed using the Cox proportional hazards model.

Supplemental Table S3: Univariate and multivariate models of overall survival

Factor	Level	N	Deaths	median	0.95LCL	0.95UCL	coef	HR	Logrank test	pvalue	CLIN coef	CLIN HR	CLIN pvalue	CYT coef	CYT HR	CYT pvalue	JOINT coef	JOINT HR	JOINT pvalue
Age							0.019	1.019	4.59	**0.032**	0.013	1.013	**0.160**	NA	NA	NA	0.015	1.015	0.116
PriorTx	No	46	34	9	3.5	23													
	Yes	27	21	3.7	2	7	0.743	2.102	6.541	**0.011**	-0.530	0.589	**0.062**	NA	NA	NA	0.908	2.479	**0.014**
Complexity	Simple	16	12	6.2	4	INF													
	Complex	57	45	3.5	3	9	0.443	1.557	1.843	0.175	0.805	2.237	**0.013**	NA	NA	NA	0.359	1.431	0.381
Cohort	MDA	38	32	3.5	2.2	7													
	OSU	37	25	9	4	24	-0.598	0.55	5.032	**0.025**	0.778	2.176	**0.031**	NA	NA	NA	-0.640	0.527	**0.035**
18q21.32 Loss	Absent	57	42	6.2	3.7	15.0													
	Present	16	15	3.2	1.8	6.5	0.963	2.620	9.548	**0.002**	NA	NA	NA	0.317	1.373	0.460	-0.137	0.872	0.788
5p14.2 Loss	Absent	52	38	6.2	4.0	15.0													
	Present	21	19	3.0	1.8	6.5	0.847	2.333	9.093	**0.003**	NA	NA	NA	0.634	1.885	**0.095**	0.953	2.595	**0.038**
11p15.3 Loss	Absent	55	42	5.0	3.5	15.0													
	Present	18	15	2.2	1.6	INF	0.798	2.221	6.591	**0.010**	NA	NA	NA	0.252	1.286	0.528	0.682	1.979	**0.110**
16q22.1 Loss	Absent	52	39	5.0	3.5	15.0													
	Present	21	18	3.4	1.6	7.0	0.662	1.939	5.026	**0.025**	NA	NA	NA	0.347	1.414	**0.350**	0.160	1.173	0.675
8p12 Gain	Absent	54	39	6.2	3.7	12.0													
	Present	19	18	2.0	1.8	15.0	0.575	1.777	4.068	**0.044**	NA	NA	NA	0.482	1.620	**0.123**	0.435	1.544	0.217
13q34 Loss	Absent	61	46	6.2	3.7	12.0													
	Present	12	11	2.6	1.8	INF	0.651	1.918	3.787	0.052	NA	NA	NA	-0.156	0.856	0.715	-0.338	0.713	0.476

* HR = hazard ratio **RED = P < 0.05** **BLUE = survives in AIC model**

CLIN = purely clinical model. CYT = Cytogenetics only model. JOINT = model that combines clinical variables with cytogenetics. Columns up to the first “pvalue” are univariate models.
0.95LCL = lower 95% confidence interval bound, 0.95UCL = upper 95% confidence interval bound.

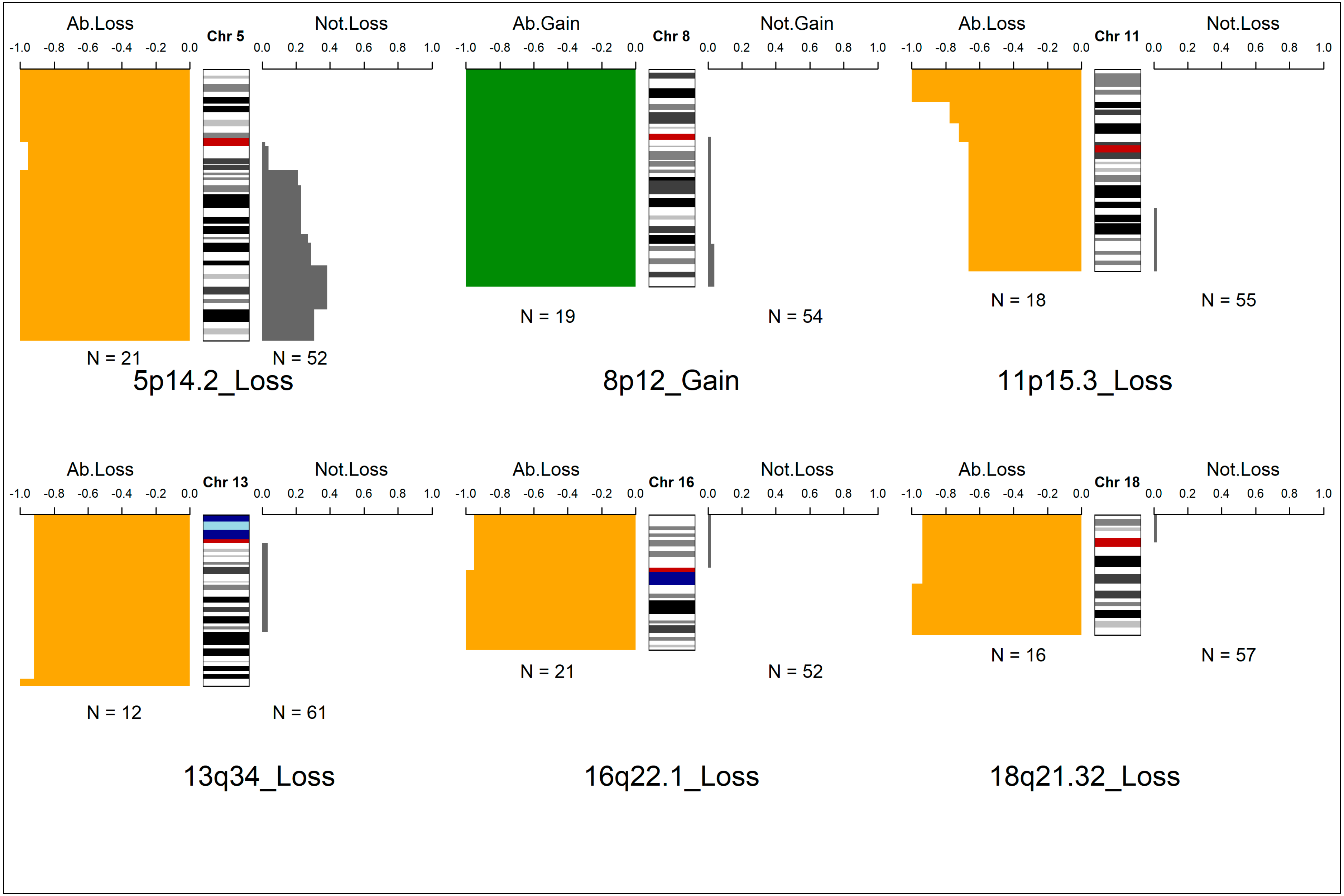

Supplemental Figure S1: Each of the six panels displays one of the six recurrent abnormalities identified as associated with overall survival in AML patients with tetraploid or near-tetraploid (T/NT) karyotypes. Each abnormality is defined by a cytogenetic event in a specific cytoband (loss of 5p14.2, gain of 8p12, loss of 11p15.3, loss of 13q34, loss of 16q22.1, or loss of 18q21.32). Each panel compares patients with that abnormality (to

the left of the ideogram; orange for loss and green for gain) to patients without (on the right, in gray). Each half-panel shows the fraction of patients with the abnormality, but displays these data for the entire chromosome. For all but one abnormality, the loss or gain fails to extend to the entire chromosome for at most one sample. The exception is a deletion of 11p that only involves the telomeric portion of the p-arm.
